# Proton stereotactic body radiotherapy for early‐stage non‐small cell lung cancer: Influence of case‐specific characteristics on dosimetric robustness

**DOI:** 10.1002/acm2.70453

**Published:** 2025-12-29

**Authors:** Akihiro Yamano, Tatsuya Inoue, Ryosuke Shirata, Masashi Yamanaka, Takayuki Yagihashi, Takahiro Shimo, Yutaro Mori, Yumiko Minagawa, Koichi Tokuuye, Weishan Chang

**Affiliations:** ^1^ Department of Medical Physics Shonan Kamakura General Hospital Kamakura Kanagawa Japan; ^2^ Department of Radiation Oncology Graduate School of Medicine Juntendo University Bunkyo Tokyo Japan; ^3^ Faculty of Medicine University of Tsukuba Tsukuba Ibaraki Japan; ^4^ Department of Radiation Oncology Shonan Kamakura General Hospital Kamakura Kanagawa Japan; ^5^ Graduate School of Human Health Sciences Tokyo Metropolitan University Arakawa Tokyo Japan

**Keywords:** dosimetric robustness, Early‐stage non‐small cell lung cancer, interplay effect, proton stereotactic body radiotherapy, robust optimization, setup uncertainties

## Abstract

**Background and purpose:**

Proton stereotactic body radiotherapy (SBRT) offers superior dose conformity. However, its clinical application remains limited due to uncertainties from setup errors and respiratory motion. This study quantified the dosimetric robustness of proton SBRT for early‐stage non‐small cell lung cancer under combined setup and interplay uncertainties and investigated the association between dosimetric robustness and case‐specific characteristics.

**Methods:**

Robust proton SBRT plans were generated considering setup and range uncertainties. Dosimetric robustness was evaluated by comparing the clinical target volume (CTV) coverage indices (V95%, V98%, and V100%) between the nominal condition and conditions incorporating combined setup and interplay uncertainties. The influence of respiratory amplitude, tumor volume, and mean tumor CT value on dosimetric robustness was assessed.

**Results:**

CTV coverage remained stable under nominal conditions. However, dose degradation occurred under combined setup and interplay uncertainties, with the median CTV V100% decreased below 70%. Larger respiratory amplitudes and lower mean tumor CT values were associated with reduced dosimetric robustness.

**Conclusions:**

Combined uncertainties significantly affected the dosimetric robustness of proton SBRT depending on case‐specific characteristics. Our exploratory analysis indicates the potential need for individualized robustness strategies in proton SBRT planning.

## 1 INTRODUCTION

Lung cancer is the leading cause of cancer‐related deaths globally, accounting for 18.7% of all cancer mortalities.^1^ Non‐small cell lung cancer (NSCLC) represents approximately 80% of lung cancers, with surgical resection as the standard treatment for early‐stage NSCLC.^2^ However, radiotherapy is a key alternative for patients ineligible for surgery due to advanced age or comorbidities.^3^ Proton therapy provides excellent dose conformity based on the Bragg peak, allowing high‐dose delivery to the tumor while sparing surrounding normal tissues. This physical benefit is particularly useful for organs affected by respiratory motion, such as the lungs and liver.^4^, ^5^, ^6^

Stereotactic body radiotherapy (SBRT) is a well‐established treatment modality that provides high local control and low invasiveness by delivering high‐dose irradiation over a few fractions. It is widely used as a standard treatment for early‐stage lung cancer in medically inoperable patients.^7^, ^8^ Proton SBRT, which applies the SBRT concept to proton therapy, was developed to further reduce normal tissue exposure. However, its clinical use remains limited due to technical challenges in dose delivery accuracy and robustness, as well as variability in implementation across facilities.^9^ According to a recent joint survey by NRG Oncology and Particle Therapy Cooperative Group, the clinical use of proton SBRT remains limited to a few advanced centers, with no established consensus on treatment strategies or fractionation schemes.^9^ Furthermore, its clinical superiority over x‐ray SBRT has not yet been clearly demonstrated.^10^ This underscores the growing need to reevaluate the clinical feasibility of proton SBRT by clarifying its dosimetric characteristics and the impact of treatment‐related uncertainties.

Due to its high‐dose, hypofractionated nature, proton SBRT is susceptible to uncertainties during delivery, such as setup uncertainties and respiratory motion. Notably, intensity‐modulated proton therapy (IMPT) is highly sensitive to the interplay effect, a temporal mismatch between beam delivery and respiratory motion that leads to dose inhomogeneity and reduced target coverage.^5^, ^6^, ^11^, ^12^, ^13^ The combined impact of setup uncertainties and interplay effects can further compromise the robustness of dose delivery.^4^, ^12^, ^13^

Although several studies have examined the effects of setup and interplay uncertainties on the robustness of conventional proton therapy, only a few reports have addressed their combined impact under proton SBRT conditions.^14^ Furthermore, no prior studies have evaluated the association between dosimetric robustness and case‐specific characteristics, such as respiratory amplitude, volume, and CT value of the target. In this study, we quantitatively evaluated the effects of setup uncertainties, interplay effects, and their combined impact on dose coverage of the clinical target volume (CTV) in IMPT‐based proton SBRT for early‐stage lung cancer. We also analyzed the relationship between case‐specific characteristics and dosimetric robustness to identify key factors contributing to compromised dose delivery.

## 2 MATERIALS AND METHODS

### 2.1 Patient data

This retrospective study utilized CT imaging data from 10 patients with early‐stage lung cancer who underwent radiotherapy at Shonan Kamakura General Hospital between March 2021 and September 2023. CT data were acquired using a Somatom Confidence scanner (Siemens Healthineers, Forchheim, Germany) with a respiratory gating system (AZ‐733 V; Anzai Medical, Tokyo, Japan). Four‐dimensional CT (4D‐CT) was performed, consisting of 10 respiratory phases (CT0–CT90%; with CT0% as end‐inhalation and CT50% as end‐exhalation), a slice thickness of 2 mm, and reconstructed resolution of 0.977 × 0.977 × 2 mm^3^. An experienced radiation oncologist delineated the gross tumor volume (GTV) in all 4D‐CT phases using RayStation 10A (RaySearch Laboratories, Stockholm, Sweden). The lungs, as organs at risk (OARs), were contoured on the CT50% phase images. To define target volumes, the internal GTV (iGTV) was generated by combining GTVs from all respiratory phases and delineating them on CT50% phase images. The CTV was created by applying a 5 mm isotropic margin to the iGTV, then cropped to remain within the lung parenchyma. Only cases with respiratory tumor motion ≥ 5 mm were included to allow adequate evaluation of the interplay effect. Tumor motion amplitude was calculated as the maximum 3D distance of the GTV centroid across the 10 respiratory phases on 4D‐CT. Table 1 summarizes tumor characteristics. Motion amplitudes ranged from 6.1 to 19.5 mm (median: 8.6 mm). CTV volume at CT50% ranged from 6.2 to 63.5 cc (median: 21.9 cc), and CTV mean HU ranged from –673.8 to –373.4 HU (median: –490.5 HU).

**TABLE 1:** Patient characteristics: Tumor location, tumor amplitude, tumor volumes, tumor mean CT values.

Patient case	Tumor location	Amplitude (mm)	CTV volume [cc]	CTV mean HU [HU]
P01	RLL	19.5	20.4	−673.8
P02	RUL	12.9	57.7	−488.6
P03	LLL	12.2	6.2	−609.7
P04	RUL	11.3	13.0	−671.5
P05	RLL	8.9	22.7	−492.4
P06	LLL	8.4	21.1	−422.7
P07	RUL	8.0	42.8	−373.4
P08	LUL	7.9	15.1	−609.3
P09	LLL	7.1	54.6	−460.0
P10	LLL	6.1	63.5	−428.2

Abbreviations: LLL, left lower lobe; LUL, left upper lobe; RUL, right upper lobe; RML, right middle lobe; RLL, right lower lobe.

This study was approved by the Institutional Review Board of Shonan Kamakura General Hospital (Tokushukai Group Ethics Committee, approval number: 2246). All procedures followed relevant guidelines and regulations. Considering the retrospective nature of the study, the ethics committee waived the requirement for written informed consent, and an opt‐out option was provided via the hospital's official website (Tokushukai Group Ethics Committee: https://www.mirai‐iryo.com/service/index.php#s03).

### 2.2 Proton SBRT planning

The VQA treatment planning system was used for proton SBRT planning and was compatible with the PROBEAT‐M1 proton delivery system (Hitachi, Tokyo, Japan), which employs a synchrotron‐based spot‐scanning technique. Beam energy ranged from 70.2 to 228.7 MeV, with penetration depths of 4 to 32 g/cm^2^. The spot size in air at the isocenter ranged from 2.5 to 7.0 mm, and the maximum field size was 30 × 40 cm^2^. For each patient, a two‐field coplanar IMPT plan was generated based on CT50% phase images, referred to as baseline static irradiation plans (static only: S0). Gantry angles were selected based on case‐specific anatomy to minimize dose to the contralateral lung and spinal cord while keeping the beam path to the target as short as possible. Layer‐wise rescanning was used to mitigate interplay effects^15^, ^16^ evenly dividing the spot dose across four rescans per isoenergy layer to average dose variation from respiratory motion.

Robust optimization used a worst‐case minimax approach, commonly referred to as the worst‐case optimization (WCO) method, with eight uncertainty scenarios, accounting for setup uncertainties of ± 5 mm in lateral, longitudinal, and vertical directions, and range uncertainties of ± 3.5%.^6^, ^11^ The prescribed dose was 42 Gy (relative biological effectiveness [RBE], RBE value: (1.1) in four fractions, normalized so that D95% of the CTV received the prescribed dose. This fractionation aligns with the standard x‐ray SBRT protocol for early‐stage lung cancer.^17^ Optimization also minimized lung and spinal cord doses. Dose calculations used a pencil beam algorithm with a triple Gaussian kernel model^18^ and a 2 × 2 × 2 mm^3^ grid resolution. The triple Gaussian model, comprising a core component (multiple Coulomb scattering) and a halo component (inelastic and elastic nuclear interactions), improves dose calculation accuracy in spot‐scanning proton therapy. Studies have shown that it closely reproduces Monte Carlo results, particularly for complex geometries such as the lungs.^18^

### 2.3 Simulation of setup uncertainties, range uncertainty, interplay effects, and their combined effects

To assess setup uncertainties, S0 plans with isocenter shifts of ± 3 and ± 5 mm in the lateral, longitudinal, and vertical directions were recalculated. For each shift magnitude, six scenarios were generated by combining positive and negative displacements along all three axes.

Static + 3 mm shift (S3): Six scenarios with ± 3 mm shifts
Static + 5 mm shift (S5): Six scenarios with ± 5 mm shifts

Range uncertainty was evaluated by recalculating S0 with relative stopping power (RSP) perturbations of +3.5% and –3.5%, generating two scenarios.

Static + 3.5% range uncertainty (R3.5): Two scenarios with ± 3.5% RSP perturbations

Next, the interplay effect was evaluated by calculating dose distribution under dynamic irradiation conditions (dynamic only: D0) using a previously developed four‐dimensional dose distribution (4DDD) tool.^6^ This tool determines each proton spot's position on each respiratory‐phase CT scan and computes the phase‐specific dose distribution based on a static plan (S0). To simulate fractionated treatment delivery, random starting phases were assigned to each fraction. The resulting phase dose distributions were accumulated on the CT50% phase images using deformable image registration^19^ to obtain the final 4DDD.

For the combined effects of interplay and setup uncertainties, the same isocenter shifts were applied to the D0 plans, generating the following.

Dynamic + 3 mm shift (D3): Six scenarios with ± 3 mm shifts
Dynamic + 5 mm shift (D5): Six scenarios with ± 5 mm shifts

In total, 28 scenarios were generated: one nominal plan (S0) and five uncertainty conditions:

S0: Static only (1 scenario, nominal plan)
S3: Static + 3 mm shift (6 scenarios)
S5: Static + 5 mm shift (6 scenarios)
R3.5: Static ± 3.5% range uncertainty (2 scenarios)
D0: Dynamic only (1 scenario)
D3: Dynamic + 3 mm shift (6 scenarios)
D5: Dynamic + 5 mm shift (6 scenarios)

**FIGURE 1:**
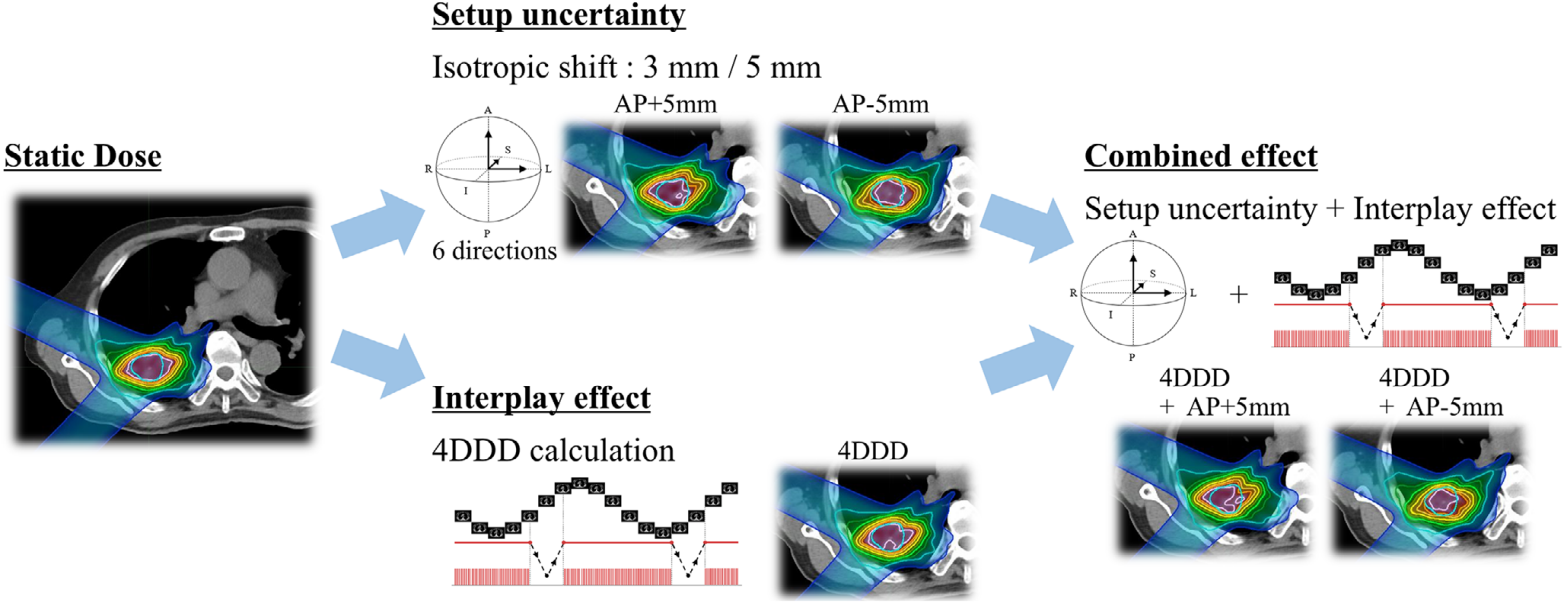
Overview of the evaluation method for combined setup uncertainty and interplay effects.

Figure 1 illustrates the planning simulation procedure for setup uncertainties, interplay effects, and combined effects.

### 2.4 Dose metrics and statistical analysis

The dose evaluation metrics included the CTV dose coverage indices (V95%, V98%, and V100%). Here, $V_{xx}$​ represents the percentage of the CTV receiving at least xx% of the prescribed dose​. For the lungs, V5 Gy, V20 Gy, and Dmean were evaluated, where V5 Gy and V20 Gy represent the percentage of lung volume receiving ≥5 Gy (RBE) and ≥20 Gy (RBE), respectively, and Dmean represents the mean lung dose. Additional OAR metrics included the Dmean for heart and esophagus, and the maximum dose (Dmax) for spinal cord and skin. These metrics were evaluated under seven irradiation conditions: one nominal (S0) and six uncertainty conditions (S3, S5, R3.5, D0, D3, and D5). Furthermore, dosimetric robustness was quantitatively assessed using difference‐based robustness metrics, defined as the change in CTV dose coverage under each uncertainty condition relative to S0. The indices were as follows.

(1) $$dV95=V95_{S0}\;-\;V95_{X}$$

(2) $$dV98=V98_{S0}\;-\;V98_{X}$$

(3) $$dV100=V100_{S0}\;-\;V100_{X}$$

where X represents the uncertainty conditions (S3, S5, R3.5, D0, D3, and D5). These difference metrics $\left(dV_{xx}\right)$​​ were used to evaluate dosimetric robustness. The relationships between tumor‐specific characteristics, respiratory amplitude, CTV volume, CTV mean HU (mean CT value of the CTV), and dosimetric robustness ($dV_{xx}$​) were analyzed using Spearman's rank correlation coefficient.

## 3 RESULTS

### 3.1 Dose distribution analysis of CTV and OARs

**FIGURE 2:**
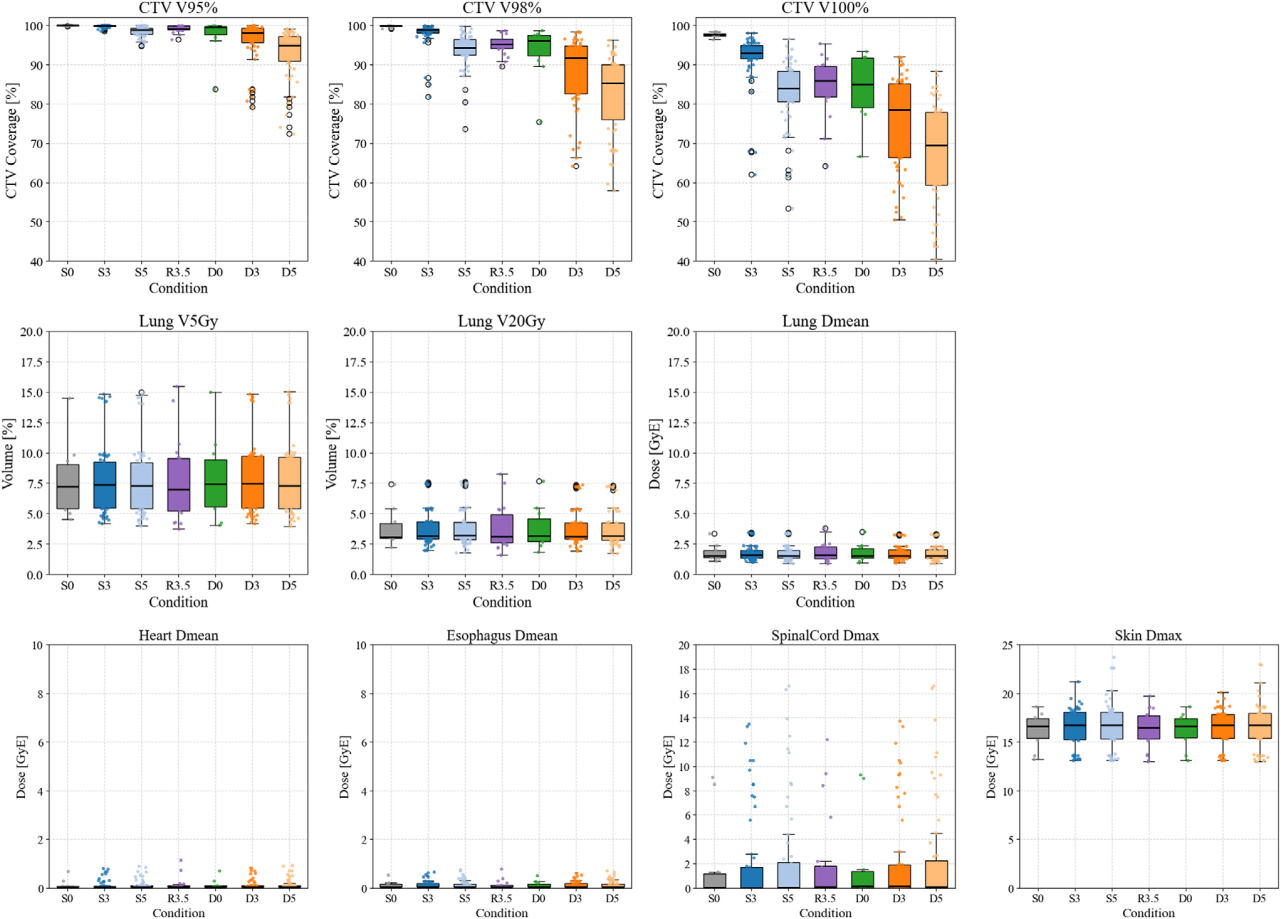
Boxplots of CTV dose coverage indices (V95%, V98%, and V100%), lung (Lungs–CTV) dose indices (V5 Gy, V20 Gy, and Dmean), and other OAR dose indices (Heart Dmean, Esophagus Dmean, Spinal Cord Dmax, and Skin Dmax) under seven irradiation conditions. S0 represents the nominal condition. S3 and S5 incorporate only setup uncertainties, with isotropic isocenter shifts of ± 3 mm and ± 5 mm, respectively. R3.5 represents a ± 3.5% range uncertainty introduced to simulate potential errors in the conversion from CT Hounsfield units to relative stopping power. D0, D3, and D5 include interplay effects, with increasing levels of combined uncertainty. CTV coverage progressively decreased with greater uncertainty, with the most pronounced reduction in V100% under D5. For the lungs and other organs at risk, all dose indices remained relatively stable across conditions, indicating minimal variation. CTV, clinical target volume; OAR, organs at risk.

Figure 2 contains boxplots of the CTV dose coverage indices (V95%, V98%, and V100%)　and lung (Lungs–CTV) dose indices (V5 Gy, V20 Gy, and Dmean), as well as additional OAR metrics (heart Dmean, esophagus Dmean, spinal cord Dmax, and skin Dmax), under seven irradiation conditions: S0, S3, S5, R3.5, D0, D3, and D5.

For the CTV, under the baseline condition (S0), CTV dose coverage was well‐maintained, with median V95%, V98%, and V100% values of 100.0%, 99.9%, and 97.6%, respectively. All patients achieved sufficient target coverage at the prescribed dose of 42 Gy (RBE). In S3, S5, and R3.5, which involved only setup uncertainty, median V95% and V98% remained above 98%, indicating preserved target coverage. However, V100% decreased to 93.0% in S3, 83.9% in S5, and 86.0% in R3.5, showing reduced full‐dose coverage. With the interplay effect included (D0, D3, D5), V100% decreased to median values of 85.0%, 78.5%, and 69.5%, respectively. V95% and V98% showed similar trends, reaching 94.9% and 85.3% in D5, indicating a marked deterioration in target dose coverage.

**FIGURE 3:**
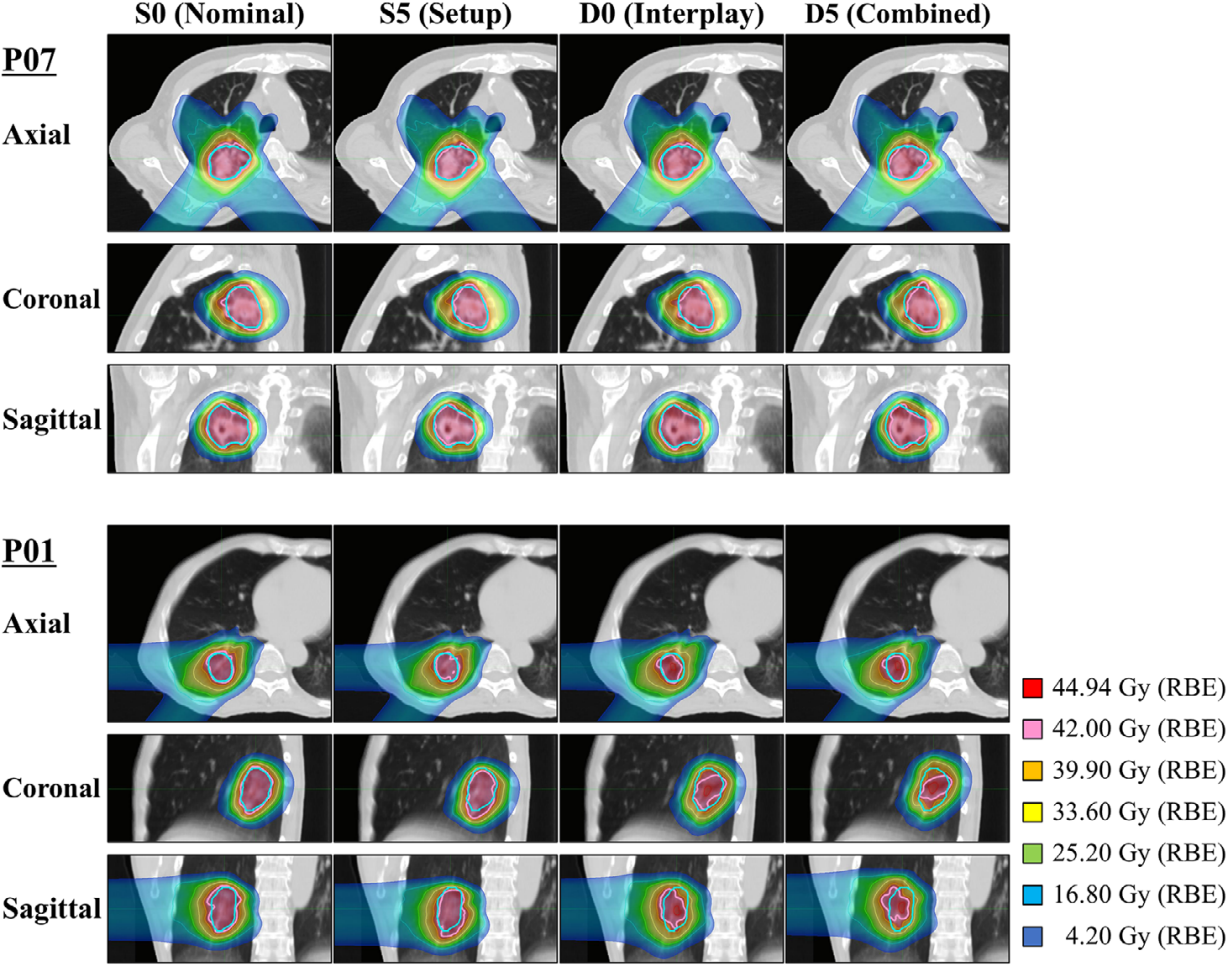
Dose distributions for the representative best‐case (P07) and worst‐case (P01) scenarios under four uncertainty conditions (S0: nominal, S5: setup, D0: interplay, D5: combined). Axial, sagittal, and coronal views are shown for each case. For S5 and D5, the simulation with the lowest CTV V100% among six realizations is displayed. The pink contour represents the 42 Gy (RBE) isodose line, and the blue contour represents the CTV boundary. BE, relative biological effectiveness; CTV, clinical target volume.

For the lungs, the dose metrics remained stable across all scenarios, with median V5 Gy ranging from 7.2% to 7.5%, V20 Gy from 3.1% to 3.2%, and Dmean from 1.54 to 1.57 Gy (RBE). No significant variation was observed, indicating OAR doses were minimally affected by uncertainties. To further illustrate the effect of uncertainties on dose coverage, two representative cases were selected. As shown in Figure 3, P01 exhibited the most severe dose degradation under D5, whereas P07 maintained favorable dose coverage across all scenarios, highlighting differences in robustness.

### 3.2 Relationship between robustness and contributing characteristics

**FIGURE 4:**
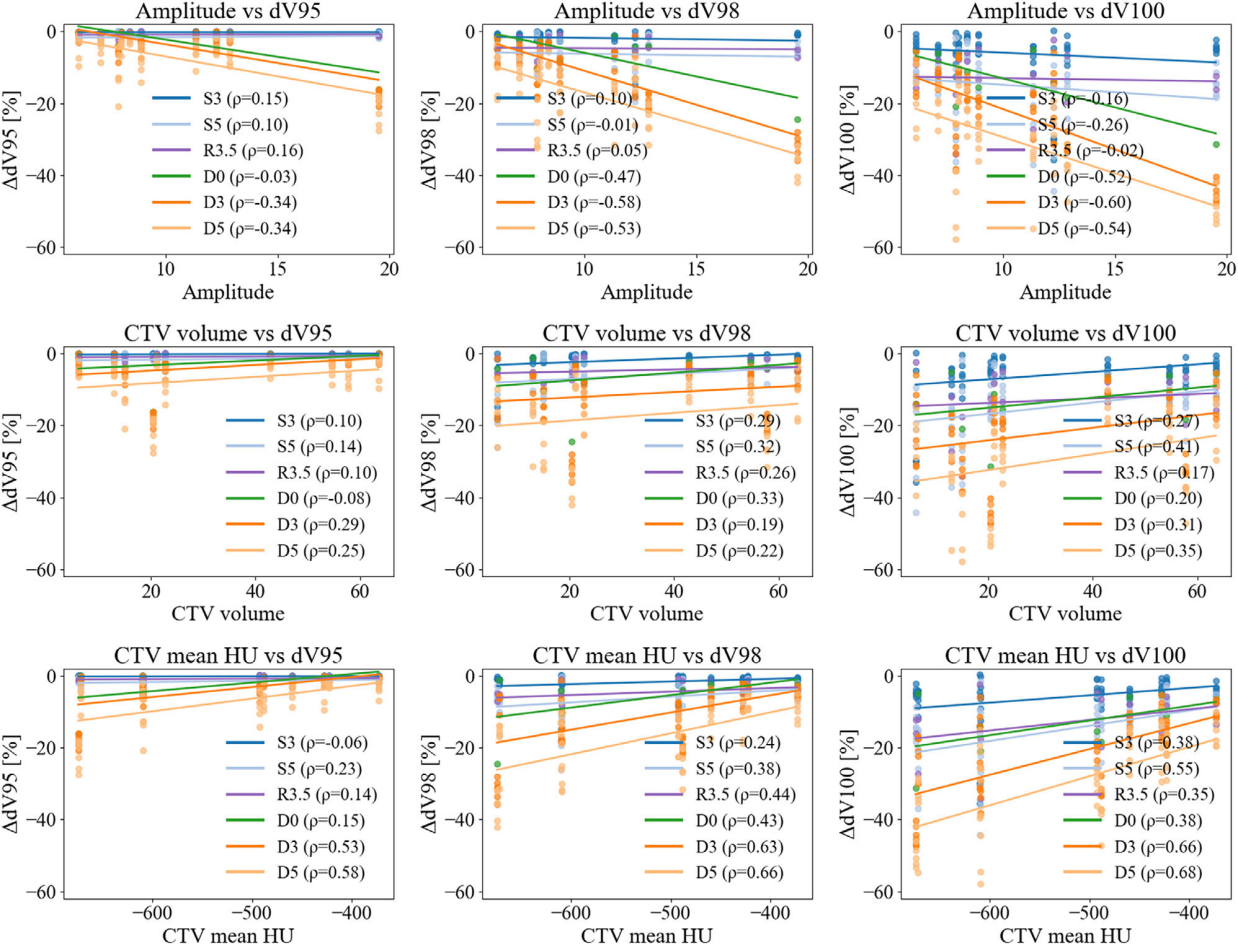
Scatter plots showing the relationship between CTV dosimetric robustness indices (dV95, dV98, and dV100) and case‐specific characteristics (amplitude, CTV volume, and CTV mean HU). Each subplot includes regression lines for six uncertainty conditions (S3, S5, R3.5, D0, D3, and D5). Image‐derived characteristics, such as respiratory amplitude and CTV mean HU, tended to correlate with robustness metrics, particularly under conditions involving interplay effects. CTV, clinical target volume.

Figure 4 presents scatter plots with regression lines illustrating the relationship between three patient‐specific characteristics (amplitude, CTV volume, and CTV mean HU) and CTV robustness metrics (dV95, dV98, and dV100) under irradiation conditions (S3, S5, R3.5, D0, D3, and D5). Amplitude showed a negative association with all robustness indices, especially under interplay‐involved conditions (D3 and D5). This was most evident under D5, where larger amplitudes corresponded to greater degradation in robustness, suggesting that tumors with larger respiratory motion were more affected by combined setup uncertainty and interplay effects. CTV volume showed a mild positive association with dV98 and dV100, mainly under setup uncertainty conditions (S3 and S5) and also under the range uncertainty condition (R3.5). The CTV mean HU generally showed a negative association with robustness metrics under interplay effect conditions. Specifically, under D5, dV100 increased in cases with lower mean CT HU, suggesting that ground‐glass opacity tumors were more susceptible to decreased dosimetric robustness. Under setup uncertainty conditions (S3 and S5) and the range uncertainty condition (R3.5), robustness metrics (dVxx) showed relatively weak associations with tumor‐specific characteristics, whereas stronger associations were observed under interplay‐effect conditions (D0, D3, and D5).

## 4 DISCUSSION

This study evaluated the robustness of proton SBRT against dose uncertainties in early‐stage NSCLC, focusing on patients with relatively large respiratory motion. To assess the reproducibility of dose delivery under realistic treatment conditions, setup uncertainties and interplay effects, two clinically relevant uncertainty sources were evaluated individually and in combination.

Under setup uncertainty conditions (S3 and S5) and the range uncertainty condition (R3.5), median V95% and V98% values remained high, indicating that the WCO method effectively compensated for geometric and range‐related perturbations. In contrast, under interplay conditions (D0, D3, and D5), V100% decreased markedly, and under D5, even V95% and V98% were substantially reduced. These results may reflect limitations of the 3D WCO method used. While WCO improves robustness through scenario‐based evaluation of geometric perturbations and is effective against setup and range uncertainties, and general anatomical variations^20^ it does not account for range changes from periodic anatomical variations due to respiratory motion. It is also unsuitable for addressing interplay effects from temporal mismatches between beam delivery and the respiratory cycle. Wei et al.^14^ reported that interplay effects in such settings can significantly compromise CTV dose coverage and that conventional robust optimization alone may be insufficient. Consistent with these findings, the present study showed that under D5, median V100% dropped below 70%, suggesting that combined uncertainties may have clinically significant effects. Recently, advanced 4D WCO methods have been proposed to incorporate temporal variations into optimization using 4DCT data.^14^, ^21^ Integrating such 4D WCO approaches may offer a promising solution to ensure dosimetric robustness in proton SBRT. However, layer‐wise rescanning with four repetitions per energy layer was applied to mitigate interplay effects, and its effectiveness depends on the relationship between the rescanning frequency, the patient's respiratory cycle, and the magnitude of tumor motion.^14^ In cases with larger respiratory amplitudes or shorter breathing periods, the temporal dose averaging achieved by four rescans may be insufficient, resulting in residual dose heterogeneity despite motion mitigation. Increasing the number of rescans or adapting the rescanning strategy based on patient‐specific motion characteristics may further improve robustness, although such approaches require additional delivery time and warrant future investigation.

The dosimetric robustness for OARs was evaluated using V5 Gy, V20 Gy, and Dmean of the lung (Lungs–CTV). These indices showed minimal variation across all uncertainty conditions, indicating consistent OAR dose maintenance. This aligns with Winter et al.’s^22^ findings, who found the mean lung dose largely unaffected by degradation from lung tissue heterogeneity. Their simulation‐based analysis suggested that the large volume and broad spatial distribution of the lung effectively averaged out localized perturbations. This supports our observation that anatomical characteristics help maintain dose stability under respiratory motion and setup errors. Proton SBRT plans are also optimized to confine high‐dose regions within the target, sparing surrounding lung tissue and preserving OAR dose metrics. Thus, in most proton SBRT cases, lung dose is inherently robust, and additional mitigation may be unnecessary. Additional OARs, including the heart, esophagus, spinal cord, and skin, were also evaluated, and these dose metrics showed only minimal variations across all uncertainty scenarios. For large target volumes or tumors near critical structures, such as the spinal cord, uncertainties’ impact on OAR doses should still be evaluated during planning.

**FIGURE 5:**
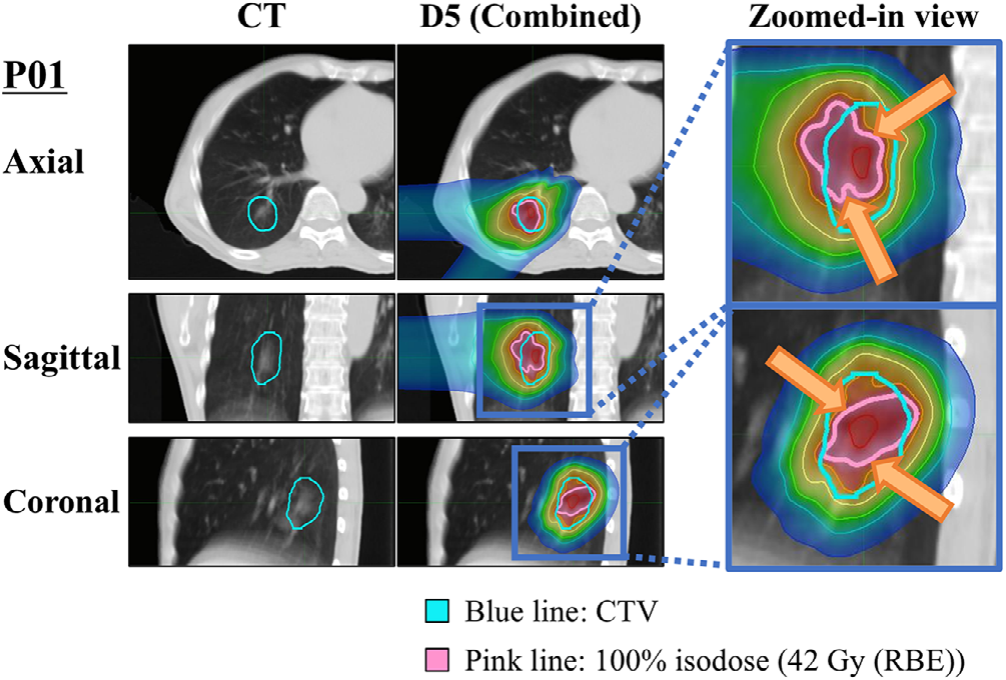
Dose distribution and cold spot formation in a ground‐glass opacity tumor (P01). CT images (left) and dose distributions under the D5 condition (combined setup and interplay uncertainties, center) are shown in axial, sagittal, and coronal views. The right panel provides zoomed‐in views highlighting spatial mismatches between the CTV (blue line) and the 100% isodose line (pink line). Cold spots observed at the distal CTV edge illustrate the potential impact of range uncertainty in low‐density tumors under interplay‐involved conditions. CTV, clinical target volume.

We investigated associations between dose robustness metrics (dV_xx) and respiratory amplitude, volume, and mean CT tumor value, to identify factors affecting robustness. A positive correlation between respiratory amplitude and difference metrics was observed, with stronger associations under interplay effects (D3, D5), suggesting that larger respiratory amplitudes reduce dosimetric robustness. This underscores the significant influence of respiratory motion on dose reproducibility in proton SBRT and aligns with previous findings.^13^ For tumor volume, mild positive correlations with dV98% and dV100% were primarily observed under setup and range uncertainties (S3, S5, R3.5). This may be attributed to the sharp distal dose falloff of proton beams beyond the Bragg peak. In smaller tumors, even slight deviations can shift part of the target beyond this gradient, creating cold spots at the distal edge. Thus, smaller tumors are more susceptible to dose degradation from setup uncertainty. Volume‐dependent sensitivity has also been reported in conventional proton therapy, particularly for small tumors where fine‐scale inhomogeneities in the lung parenchyma can degrade dose.^22^ These findings suggest tumor volume remains important in proton SBRT planning. Negative correlations between the mean tumor CT value and dV100% were observed under interplay effect conditions. Tumors with low mean CT values, such as ground‐glass opacities, show reduced dosimetric robustness. This is rarely noted in the literature and offers a novel perspective on proton SBRT robustness assessment. This may result from the physical characteristics of low‐density tissues, where the electron–mass density relationship is nonlinear, steepening the CT to RSP conversion slope and increasing RSP errors.^23^ Vestergaard et al.^24^ reported that in pulmonary regions with CT values from –700 to –400 HU, RSP errors up to 7% can occur, increasing range uncertainty, shifting the Bragg peak by several millimeters, and raising cold spot risk at the distal CTV edge. Figure 5 illustrates this, showing a ground‐glass opacity tumor with a pronounced spatial mismatch between the CTV (blue line) and 100% isodose line (pink line) under the D5 condition, highlighting the higher cold spot risk in low‐density tumors. In this study, the range uncertainty parameter in robustness optimization was uniformly set to 3.5%. However, such fixed assumptions may be unsuitable for tumors with low CT values. Adjusting robustness parameters and range settings using CT‐derived information on a case‐specific basis may provide a more practical and individualized approach to improving dosimetric robustness in proton SBRT. These findings suggest that patients with large respiratory amplitudes, small tumor volumes, or low mean tumor CT values may be more susceptible to dose degradation, highlighting the need for tailored strategies in such scenarios.

This study did not incorporate range uncertainty into the combined effect. A comprehensive dosimetric investigation will be essential in future work to more accurately characterize their interactions with respiratory motion, interplay effects, and patient‐specific factors. Although limited to 10 cases, this study showed consistent trends across multiple uncertainty conditions and case‐specific characteristics. These preliminary findings provide insight into how respiratory motion and anatomical variations influence the dosimetric robustness of proton SBRT. Case‐specific characteristics, particularly respiratory amplitude, tumor volume, and mean tumor CT value, were linked to reduced dosimetric robustness. Incorporating these factors into treatment planning may enable individualized robust optimization. Smaller tumors, which are more vulnerable to cold spot formation due to the sharp distal falloff of proton beams, may benefit from careful beam arrangement or a tailored robust margin, though further validation is needed. Similarly, tumors with low mean CT values, such as ground‐glass opacities, are more vulnerable to range uncertainties from larger CT‐to‐RSP conversion errors. In such cases, adjusting robustness parameters based on individual characteristics or using advanced techniques, such as dual‐energy CT^25^ or body composition‐based RSP estimation^26^ may improve dosimetric robustness. Future studies could develop quantitative risk stratification and classification models based on imaging characteristics to guide individualized planning, including delivery technique selection (e.g., IMPT vs. proton SBRT). Integrating real‐time monitoring could further enable adaptive proton therapy.^27^

## 5 CONCLUSION

This study quantitatively evaluated the dosimetric robustness of proton SBRT for early‐stage non‐small cell lung cancer under combined setup and interplay uncertainties. The analysis revealed that these combined uncertainties can lead to clinically relevant dose degradation, particularly in cases with large respiratory motion, small target volumes, or low mean CT values. Our exploratory analysis indicates the potential need for individualized robustness strategies in proton SBRT planning that incorporate case‐specific and image‐derived parameters such as respiratory amplitude, target size, and CT values.

## AUTHOR CONTRIBUTIONS

Akihiro Yamano and Tatsuya Inoue contributed substantially to the conception and methodology of this study. Akihiro Yamano, Tatsuya Inoue, Ryosuke Shirata, Masashi Yamanaka, and Takayuki Yagihashi contributed to data curation, analysis, and interpretation. Akihiro Yamano, Tatsuya Inoue, Yutaro Mori, Yumiko Minagawa, Koichi Tokuuye, and Weishan Chang contributed to study design and supervision. Akihiro Yamano and Tatsuya Inoue drafted the manuscript. All authors critically reviewed and revised the manuscript and approved the final version for submission.

## DECLARATION OF GENERATIVE AI IN SCIENTIFIC WRITING

During the preparation of this work, the authors used ChatGPT in order to improve the readability of the manuscript. After using this tool, the authors reviewed and edited the content as needed and take full responsibility for the content of the publication.

## CONFLICT OF INTEREST STATEMENT

The authors declare that they have no known competing financial interests or personal relationships that could have appeared to influence the work reported in this paper.

## DATA AVAILIBILITY STATEMENT

Data supporting the findings of this study are available from the corresponding author upon reasonable request.
